# A Novel Insight into Paraptosis-Related Classification and Signature in Lower-Grade Gliomas

**DOI:** 10.1155/2022/6465760

**Published:** 2022-11-14

**Authors:** Xi-Feng Qian, Jia-Hao Zhang, Yue-Xue Mai, Xin Yin, Yu-Bin Zheng, Zi-Yuan Yu, Guo-Dong Zhu, Xu-Guang Guo

**Affiliations:** ^1^Department of Clinical Laboratory Medicine, The Third Affiliated Hospital of Guangzhou Medical University, Guangzhou 510150, China; ^2^Department of Clinical Medicine, The Sixth Clinical School of Guangzhou Medical University, Guangzhou 511436, China; ^3^Department of Pediatrics, The Pediatrics School of Guangzhou Medical University, Guangzhou 511436, China; ^4^Department of Clinical Medicine, The Third Clinical School of Guangzhou Medical University, Guangzhou 511436, China; ^5^Department of Oncology, Guangzhou Geriatric Hospital, Guangzhou 510180, China; ^6^Department of Geriatrics and Oncology, Guangzhou First People's Hospital, Guangzhou 510180, China; ^7^Key Laboratory for Major Obstetric Diseases of Guangdong Province, The Third Affiliated Hospital of Guangzhou Medical University, Guangzhou 510150, China; ^8^Key Laboratory of Reproduction and Genetics of Guangdong Higher Education Institutes, The Third Affiliated Hospital of Guangzhou Medical University, Guangzhou 510150, China

## Abstract

Lower-grade gliomas (LGG) are the most common intracranial malignancies that readily evolve to high-grade gliomas and increase drug resistance. Paraptosis is defined as a nonapoptotic form of programmed cell death, which is gradually focused on patients with gliomas to develop treatment options. However, the specific role of paraptosis in LGG and its correlation is still vague. In this study, we first establish the novel paraptosis-based prognostic model for LGG patients. The relevant data of LGG patients were acquired from The Cancer Genome Atlas database, and we found that LGG patients could be divided into three different clusters based on paraptosis via consensus cluster analysis. Through least absolute shrinkage and selection operator regression analysis and multivariate Cox regression analysis, 10-paraptosis-related gene (PRG) signatures (CDK4, TNK2, DSTYK, CDKN3, CCR4, CASP9, HSPA5, RGR, LPAR1, and PDCD6IP) were identified to separate LGG patients into high- and low-risk subgroups successfully. The Kaplan–Meier analysis and time-dependent receiver-operating characteristic showed that the performances of predicting overall survival (OS) were dramatically high. The parallel results were reappeared and verified by using the Chinese Glioma Genome Atlas and Gene Expression Omnibus databases. Independent prognostic analysis and nomogram construction implied that risk scores could be considered the independent factor to predict OS. Enrichment analysis indicated that immune-related biological processes were generally enriched, and different immune statuses were highly infiltrated in high-risk group. We also confirmed the potential relationship of 10-PRG signatures and drug sensitivity of Food and Drug Administration–approved drugs. In summary, our findings provide a novel knowledge of paraptosis status and crucial direction to further explore the role of PRG signatures in LGG.

## 1. Background

Gliomas are the most common intracranial malignancies in the range of primary central nervous system tumors [1]. According to the World Health Organization classification criteria, lower-grade gliomas (LGG) consist of grade II and III neoplastic lesions, histologically including astrocytomas, oligoastrocytomas, and oligodendrogliomas [2, 3]. As the diffuse invasive tumors, LGG with high variability are arduous to predict the clinical course, which is further aggravated by the subjective variability of the operator in charge of histologic classification and grading [4]. In the management for LGG, optimal surgical resection allows for diagnostic and therapeutic resection while minimizing side effects from excessive neurological deficits [5]. Radiotherapy remains the standard treatment for LGG postoperatively and poor outcomes without this; besides, adjuvant chemotherapy can prolong progression-free survival and overall survival (OS) in these patients [5]. Currently, the lack of effective diagnostic strategies, which relies on neurological and neuroimaging tests performed at an advanced stage of the disease, is one of the major problems leading to poor management of gliomas [6]. Besides, due to the great internal heterogeneity in tumor biological behavior, LGG readily and inevitably evolve to high-grade gliomas and increasing drug resistance, leading to the sustained high mortality [7, 8]. Glioma has characterized a series of DNA mutations and disorder of non-coding RNA. The circulating small molecules found in blood and other biological fluids, such as miRNA and protein related to circulating tumor cells or intracellular and extracellular vesicles, may be used as markers for early diagnosis and classification of brain tumors [9]. Some tumor biomarkers including IDH mutation, 1p/19q codeletion, p53 mutation, and MGMT promoter methylation had been studied as predictive significance and could be biomarker-guided predictors to predict prognosis and guide treatment in LGG patients [3, 7, 10]. Although several candidate biomarkers were unearthed, a few of these are applied as diagnostic or prognostic markers in clinical routines and hitherto none of which are used as an approach for preliminary diagnosis [3]. Identifying novel effective biomarkers for LGG is greatly required.

Paraptosis, defined as a nonapoptotic alternative form of programmed cell death, is different from apoptosis in terms of morphology, biochemistry, and the response of apoptosis inhibitors. Paraptosis presents as extensive cytoplasmic vacuolation and organelle swelling that begins with endoplasmic reticulum (ER) dilation and vacuolation, alongside mitochondria swelling and fusion [11]. The progression of paraptosis does not involve the activation of caspases and the formation of apoptotic bodies; also, it is non-responsive to apoptotic inhibitors and requires protein synthesis [11, 12]. The program of paraptosis also might be induced by IGFIR triggering two paraptosis signaling pathways, MAPK/ERK and JNK/SAPK2, while AIP1/Alix could inhibit IGFIR activity [12]. Additionally, researchers observed that paraptosis also occurred in the Zika virus-infected cells via PI3K/AKT signaling axis [13]. ER stress could launch the program of apoptosis and lead to extensive ER-derived vacuoles to trigger paraptosis-like death in the event of the incessancy of ER stress [14]. Ca^2+^ transport has been proved to be significant in paraptosis induction, particularly in the interaction between ER and mitochondria [15]. Paraptosis is also regulated by p53 as well as necroptosis, ferroptosis, pyroptosis, and other non-classical cell death pathways; however, the different pathways possess different regulated proteins [16]. In addition, several anticancer drugs, such as curcumin, celastrol, 15d-PGJ2, ophiobolin A, and paclitaxel, have proved their anti-cancer function by inducing paraptosis-relative cell death [17]. Moreover, Ghosh et al. found that withaferin A induced reactive oxygen species (ROS)-mediated paraptosis to cause cancer cell death in two cell lines of human breast cancer [18]. With regard to gliomas, Chen et al.'s experiments discovered that polymorphonuclear leukocytes and macrophages were involved to kill T9-C2 glioma cells through paraptosis-induced program in Fischer rats [19]. Researchers have found that the expression of paraptosis-related genes (PRGs) is closely related to the malignancy of glioma. Ophiobolin A can disrupt the homeostasis of internal potassium ion and curcumin affected the integrity of the reticulum, which together induces paraptosis-like cell death in human glioblastoma cells [20, 21]. Based on the above, paraptosis-induced expression has significant effect on the tumorigenesis and malignant progression. However, the specific prognostic role of paraptosis in LGG is still vague.

Extensive sequencing technologies, such as gene chip and high-throughput sequencing, have been utilized over the past decade. The expression profile of all genes by using above methods that can quickly detect within the same sample time point, is particularly eligible for screening out differentially expressed genes [22]. However, owing to the heterogeneity of tissue or sample and high false alarm rate in independent studies, the outcomes are always limited, inconsistent, or unpersuasive [23]. Integrating and re-analyzing these expression profile can provide valuable clues for new study. For this purpose, comprehensive bioinformatics analyses were qualified to provide novel insights on the regulation of PRGs in LGG patients in this study. Besides, this computational method was conducive to the potential finding to identify new epigenetic markers for diagnosis and prognosis and specific targets for cancer therapeutics [24]. In this study, we first established and validated the novel paraptosis-based subtypes and prognostic scoring model for LGG patients.

## 2. Results

### 2.1. Confirmation of DE-PRGs

**Figure 1:**
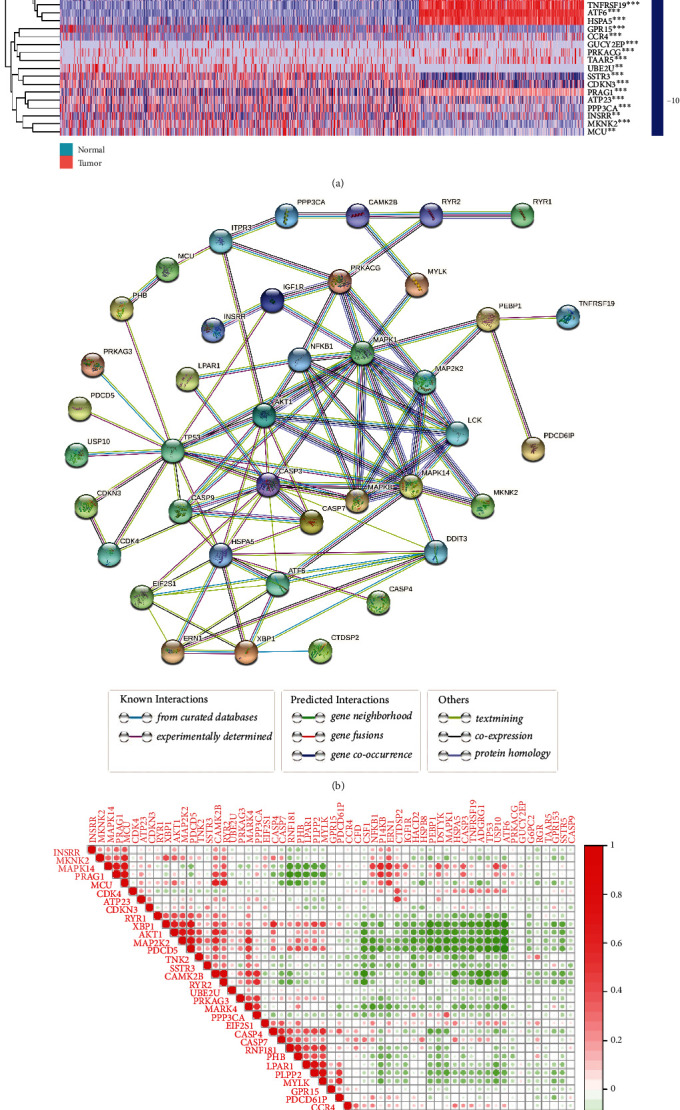
Expressions of the 59 DE-PRGs and the interaction network of PRGs. (a) Heatmap (green represents low expression level; red represents high expression level) of the DE-PRGs between the normal (N, brilliant blue) and the tumor tissues (T, red). *P* values are shown as: ∗∗*P* < 0.01; ∗∗∗*P* < 0.001. (b) PPI network showing the interactions of the PRGs (interaction score = 0.7 and hidden disconnected nodes). (c) The correlation between PRGs (red represents positive correlation; green represents negative correlation).

1152 normal samples and 529 LGG samples were included from Genotype-Tissue Expression (GTEx) and The Cancer Genome Atlas (TCGA) databases, respectively. 66 PRGs were selected to further analyze between tumor and normal samples. Then, 59 differentially expressed PRGs (DE-PRGs) were identified, and their expression levels were exhibited in Figure 1(a). In detail, there were 33 DE-PRGs upregulated, whereas 26 DE-PRGs downregulated in tumor tissue compared with normal tissue. Additionally, a protein–protein interaction (PPI) network was conducted to confirm the hub genes, and the results were as follows: MAPK1, TP53, CASP3, HSPA5, MAPK14, AKT1, ATF6, DDIT3, MAPK8, and NFKB1 (Figure 1(b)). The intrinsic connection of DE-PRGs was presented to find the regulation situation between them (Figure 1(c)).

### 2.2. Paraptosis-Related Analysis in Clusters

**Figure 2:**
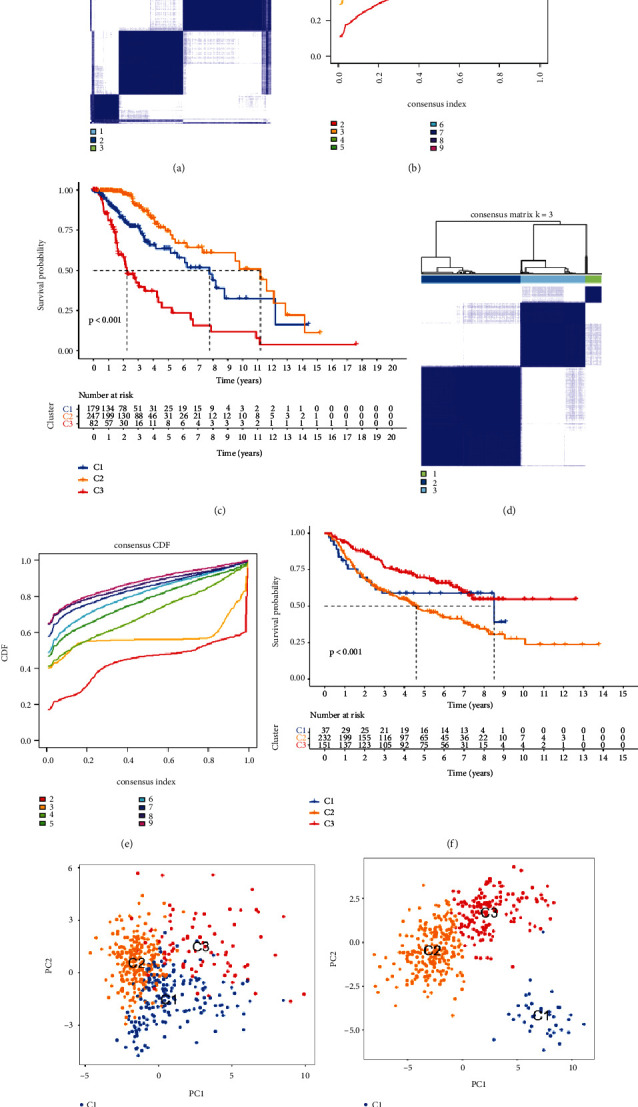
LGG clustering based on PRGs. LGG patients divided into three clusters according to the consensus clustering matrix in TCGA (a) and CGGA (d) datasets. Empirical cumulative distribution function plot in TCGA (b) and CGGA (e) datasets. Kaplan–Meier curves for the three clusters in TCGA (c) and CGGA (f) datasets. PCA for two clusters in TCGA (g) and CGGA (h) datasets.

**Figure 3:**
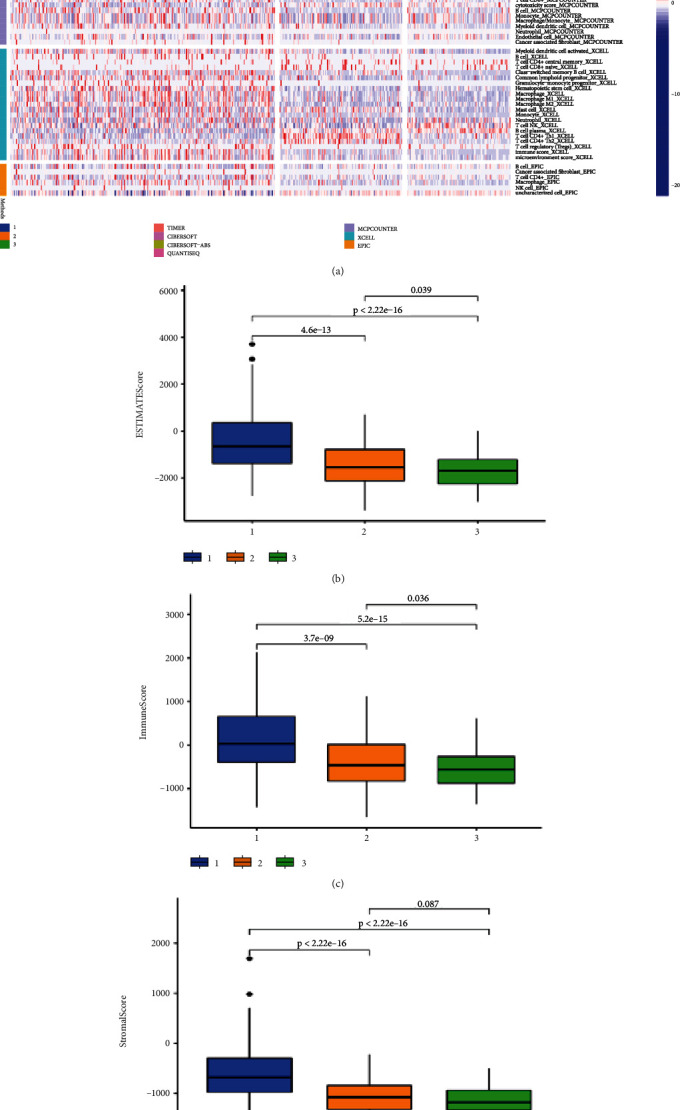
The immune infiltration divisions of paraptosis response-related clusters in LGG based on TCGA cohort. (a) The immune cell analysis of clusters in multi-immune algorithms. (b–d) The contrasts of ESTIMATE score, stromal score, and immune score among the three clusters.

To further reveal the similarity and difference between gene expression levels, we conducted the consensus clustering analysis. Results found that there was a better distinction significant when *k* = 3 with a curve of gentle slope; therefore, LGG patients can be divided into three clusters in both TCGA and Chinese Glioma Genome Atlas (CGGA) cohorts (Figure 2(a), 2(b), 2(d), and 2(e)). Besides, the results of principal-component analysis (PCA) also proved that either cluster 1, cluster 2, or cluster 3 could be concentrated in a specific area, and the three clusters could be well separated (Figures 2(g) and 2(h)). Subsequently, we found that there was significant difference in OS of three clusters in two cohorts (Figures 2(c) and 2(f)). Probing immune infiltration in paraptosis subtypes probably gives potential insights for immunotherapy. The heatmap displayed that immune enrichment was preferentially relevant to LGG subtypes by combining analysis with 7 algorithms (*P* < 0.05; Figure 3(a)). For example, macrophage infiltration was up-regulated in cluster 1, whereas B cell infiltration was down-regulated in cluster 3, and so on. In addition, the ESTIMATE score, immune score, and stromal score were higher in cluster 1 and lower in cluster 3 (most *P* < 0.05; Figure 3(b)–3(d)). All in all, cluster analysis founded on PRGs has a fabulous classification and significance for the LGG patients.

### 2.3. Establishment of Prognostic Model

**Figure 4:**
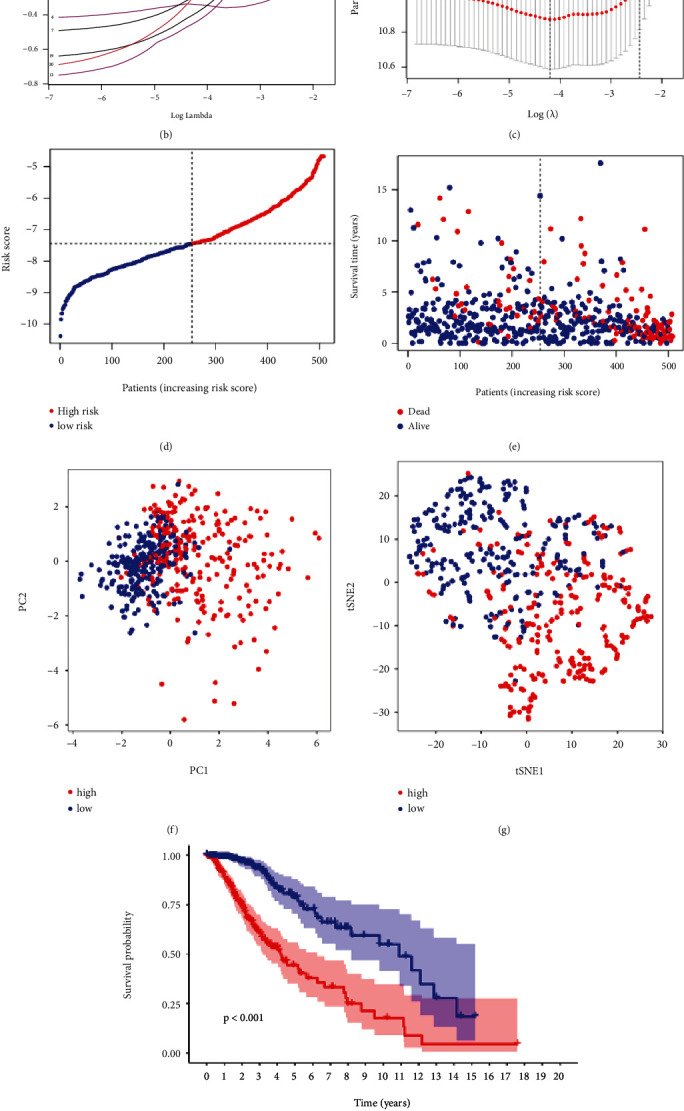
Construction of risk signature in the TCGA cohort. (a) Univariate Cox regression analysis of OS for DE-PRGs. (b) LASSO regression of the 14 OS-related genes. (c) Cross-validation for tuning the parameter selection in the LASSO regression. (d) Distribution of patients based on the risk score. (e) The survival status for each patient (low-risk population: on the left side of the dotted line; high-risk population: on the right side of the dotted line). (f) PCA plot for LGG patients based on the risk score. (g) *t*-SNE plot for LGG patients based on the risk score. (h) Kaplan–Meier curves for the OS of patients in the high- and low-risk groups. (i) ROC curves demonstrated the predictive efficiency of the risk score.

A prognostic model was established to probe the role of PRGs in LGG. Through performing univariate Cox regression analysis, 20 PRGs were defined as the candidate genes (Figure 4(a)). Subsequently, least absolute shrinkage and selection operator (LASSO) analysis further narrowed the candidate genes and screened 14 PRGs when having optimal *λ* values (Figures 4(b) and 4(c)). Afterwards, 10 PRG signatures (CDK4, RGR, TNK2, LPAR1, DSTYK, CCR4, CDKN3, PDCD6IP, CASP9, and HSPA5) were finally identified with multivariate Cox regression model. As a result, we constructed the risk score, which can be calculated by: risk score = (0.219 × CDK4 expression) + (−0.329 × RGR expression) + (−0.515 × TNK2 expression) + (0.144 × LPAR1 expression) + (−0.614 × DSTYK expression) + (0.617 × CCR4 expression) + (0.323 × CDKN3 expression) + (0.351 × PDCD6IP expression) + (−0.682 × CASP9 expression) + (−0.666 × HSPA5 expression). According to the median risk score of 10-PRG signatures, the TCGA patients were divided into high-risk (*n* = 254) and low-risk (*n* = 254) subgroups, respectively (Figure 4(d)). The plot of the high- and low-risk groups in terms of survival time and status demonstrated that the higher the risk score, the greater mortality, and poorer survival outcome (Figure 4(e)). The PCA and *t*-distributed stochastic neighbor embedding (*t*-SNE) analyses can be used to verify whether LGG patients were predominantly grouped based on the risk score and signatures' expression. The results indicated that they sorted the patients into two obvious and different discrete directions (Figures 4(f) and 4(g)). Moreover, Kaplan–Meier curve illustrated that the high-risk patients have poor life span (*P* < 0.001; Figure 4(h)). Thus, the risk classification has a great considerable significance. Meanwhile, the result of time-dependent receiver-operating characteristic (ROC) analysis showed that area under the ROC curve (AUC) was predicted as 0.889, 0.866, and 0.794 for 1-, 3-, and 5-year OS, respectively (Figure 4(i)). Next, GEPIA2—a bioinformatics resource database, can do further analysis to prove the prognostic value in depth. The survival plots signified that the prognostic conditions of 10 PRGs were nearly consistent with univariate results (Figure S1).

### 2.4. Validation of 10-PRG Signatures

**Figure 5:**
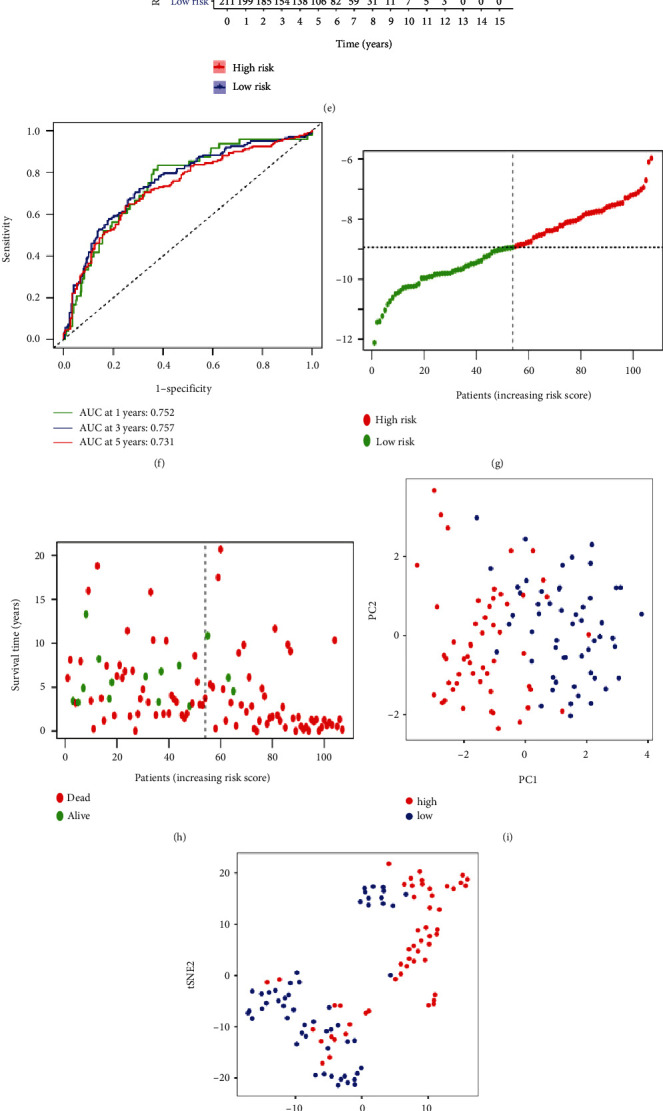
Validation of the risk model in the CGGA (a)–(f) and GEO (g)–(l) cohorts. (a) and (g) Distribution of patients based on the risk score. (b) and (h) The survival status for each patient (low-risk population: on the left side of the dotted line; high-risk population: on the right side of the dotted line). (c) and (i) PCA plot for LGG patients based on the risk score. (d) and (j) *t*-SNE plot for LGG patients based on the risk score. (e) and (k) Kaplan–Meier curves for the OS of patients in the high- and low-risk groups. (f) and (l) ROC curves demonstrated that the predictive efficiency of the risk score.

To validate whether the prognostic model was stable and authentic, we operated the similar analyses in the external datasets from CGGA and Gene Expression Omnibus (GEO) databases. The same risk score formula was used to calculate the risk score and stratified them into high-risk (*n* = 209) and low-risk (*n* = 211) subgroups in CGGA, and *n* = 53 and *n* = 54 in GEO, respectively (Figures 5(a) and 5(g)). The patients with low-risk scores had lower deaths and longer survival time (Figures 5(b) and 5(h)). Similarly, PCA and *t*-SNE analyses showed obvious distribution of two groups (Figure 5(c), 5(d), 5(i), and 5(j)). The survival curve was presented in Figures 5(e) and 5(k), indicating that the higher survival probability of low-risk group and a better statistical significance were found between two groups (*P* < 0.01) in two cohorts. Following this, the ROC curve showed that the predicted values of AUC of 1-, 3-, and 5-year OS were 0.752, 0.757, and 0.731 in CGGA cohort, and 0.825, 0.807, and 0.745 in GEO cohort, respectively (Figures 5(f) and 5(l)). In summary, the above descriptions of CGGA and GEO cohorts are exceedingly consistent with the TCGA cohort, which further means that the prognostic risk model is reliable and effective.

### 2.5. Independent Analysis of 10-PRG Prognostic Model

**Figure 6:**
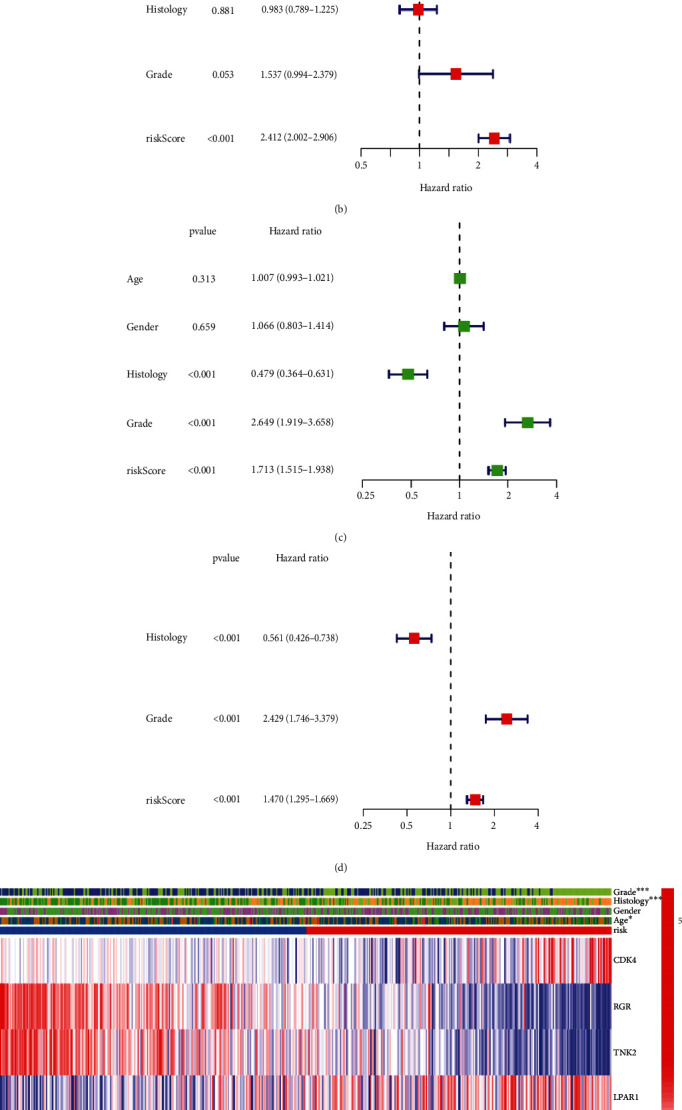
Univariate and multivariate Cox regression analyses for the risk score. Univariate analysis for the TCGA (a) and CGGA (c) datasets. Multivariate analysis for the TCGA (b) and CGGA (d) datasets. (e) Heatmap (green represents low expression level; red represents high expression level) for the connection between clinical characteristic and risk scores. *P* values are showed as: ∗*P* < 0.05; ∗∗∗*P* < 0.001.

It is necessary to further analyze whether the risk model is regarded as a prognostic factor independent of other factors. In TCGA cohort, the univariate Cox analysis implied that the age, grade, histology, and risk score had significant statistical significances, whereas the results of multivariate Cox analysis presented that only the age and the risk score were regarded as the independent prognostic factors (*P* < 0.001; HR: 1.054 and 2.412, respectively; Figures 6(a) and 6(b)). In the CGGA cohort, according to the same clinical category information, the multivariate Cox regression results disclosed that grade, histology, and risk score could be used as independent prognostic factors (*P* < 0.01; HR: 2.429, 0.561, and 1.470, respectively; Figure 6(c) and 6(d)). Hence, this risk score can be used as the independent prognostic factor in this model. In addition, the heatmap of clinical features connected with the expression level of 10-PRG signatures was shown that there were distinguishable differences in grade, histology, and age (Figure 6(e)).

### 2.6. Construction and Validation of Nomogram

**Figure 7:**
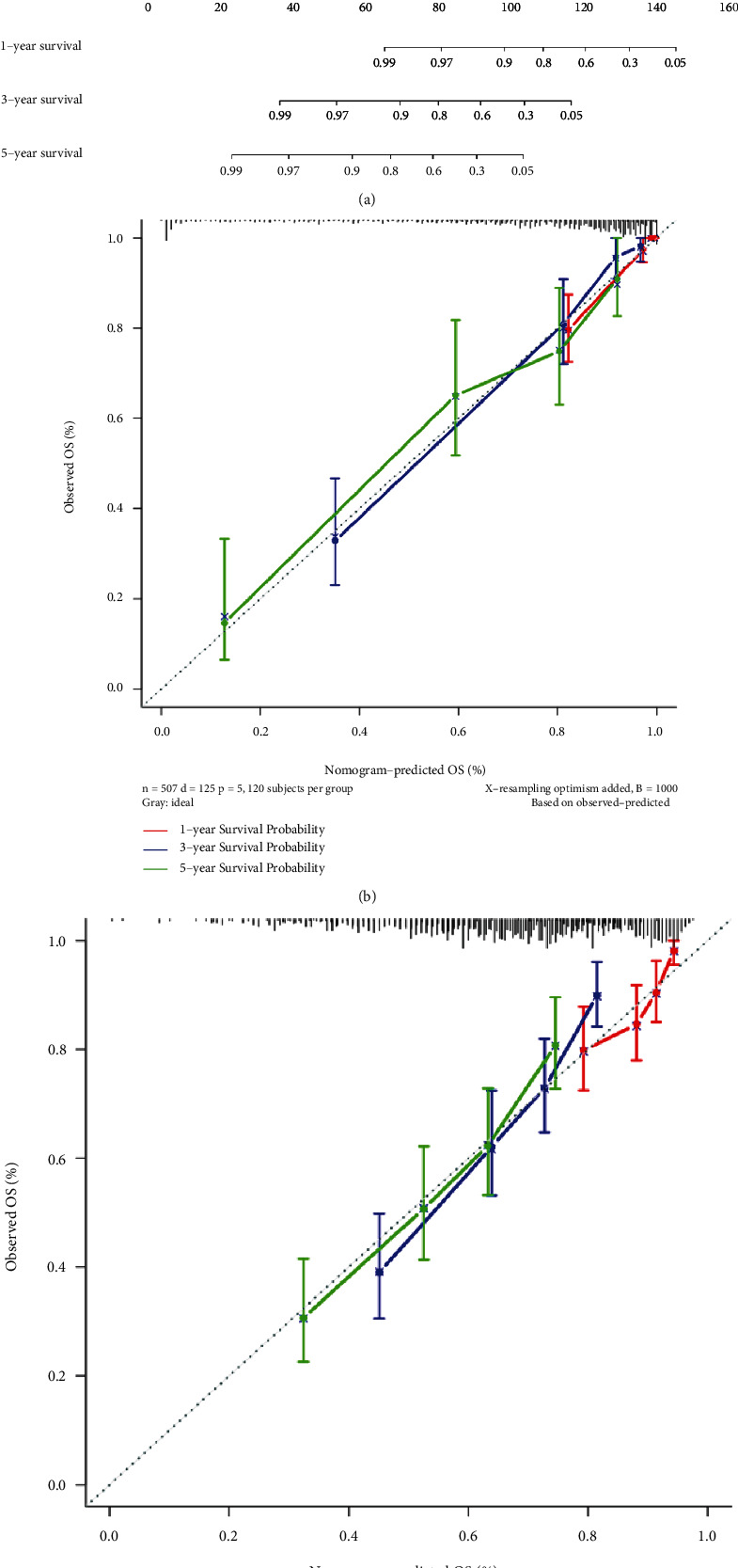
Clinical prognostic nomogram for survival prediction. (a) Clinical prognostic nomogram was developed to predict 1-, 3-, and 5-year survival times. (b) Calibration curves of nomograms in the TCGA cohort. (c) Calibration curves of nomograms in the CGGA cohort.

Nomogram can comprehensively analyze the various factors to exhibit OS-related information combined with clinical features based on the gene expression of patients. Thus, nomogram that combined with various clinical information was constructed to provide a scoring system to predict 1-, 3-, and 5-year OS possibilities of the LGG patients (Figure 7(a)). Additionally, the calibration curves evinced perfect accuracy for the nomogram model to predict the prognosis of LGG patients in the TCGA and CGGA cohorts (Figures 7(b) and 7(c)). In addition, *C*-index of the nomogram was 0.865 in TCGA and 0.691 in CGGA. Therefore, the combination of clinical characteristics and risk scores based on 10-PRG signatures showed outstanding prognostic value of the LGG patients.

### 2.7. Functional and Pathway Enrichment Analyses

**Figure 8:**
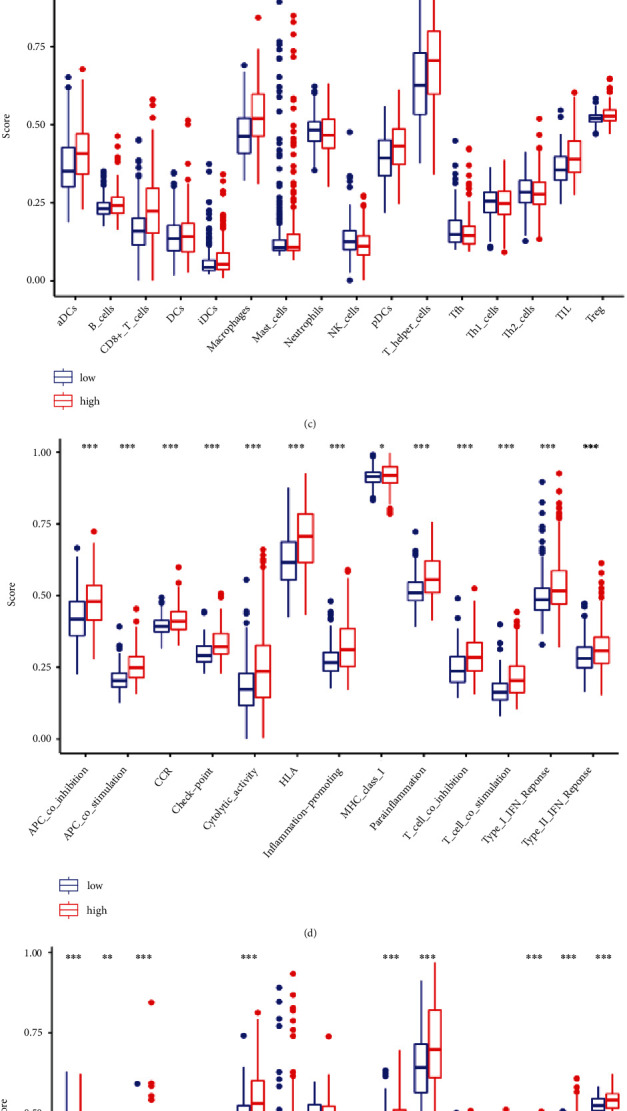
Functional analysis and immune related analysis. (a) Bubble graph for GO enrichment (the bigger bubble means the greater genes enriched, and the increasing depth of red stands for the differences being more obvious; *q*-value: the adjusted *P*-value). (b) Bar plot for KEGG pathways (the longer bar equals the more genes enriched, and the increasing depth of red means the differences were more obvious). Comparison of the enrichment scores of 16 types of immune cells between low- (blue box) and high-risk (red box) groups in TCGA (c) and CGGA (e) datasets. Comparison of the enrichment scores of 13 immune-related pathways between low- (blue box) and high-risk (red box) groups in TCGA (d) and CGGA (f) datasets.

In analyzing the biological functions, Gene Ontology (GO) analysis based on these DE-PRGs of two risk groups suggested that the DE-PRGs were chiefly enriched in antigen processing and presentation via MHC class II in biological process, MHC-related protein complex in cellular component, and MHC class II receptor activity, peptide antigen binding, and immune receptor activity in molecular function (Figure 8(a)). The results of GO analysis further denoted that PRGs are active in antigen presentation processes and MHC biological processes. Kyoto Encyclopedia of Genes and Genomes (KEGG) results illustrated that these PRGs were associated with the immune-related and other diseases-related pathways: antigen processing and presentation, Th1 and Th2 cell differentiation, Th17 cell differentiation, TGF-*β* signaling pathway, systemic lupus erythematosus, and tuberculosis (Figure 8(b)). The GO and KEGG analyses indicate that paraptosis is closely correlated with immunity.

### 2.8. Immune Correlation Analysis

To explore the immune-related mechanism in LGG, single-sample Gene Set Enrichment Analysis (ssGSEA) was conducted in the enrichment scores of 16 immune-related cells and 13 pathways analyses. The results displayed that the aDC cells, B cells, CD8^+^ T cells, iDC cells, macrophages, pDC cells, T helper cells, Tfh, TIL cells, and Treg cells were significantly enriched in high-risk group (TCGA cohort). However, NK cells were enriched more in low-risk group (*P* < 0.001) (Figure 8(c)). In CGGA cohort, the immune cells in high-risk group were generally enriched, especially the aDC cells, B cells, CD8^+^ cells, macrophages, T-helper cells, pDC cells, Th2 cells, TIL cells, and Treg cells (*P* < 0.001; Figure 8(e)). All immune-related pathways showed more apparent activity in high-risk group of TCGA and CGGA cohorts than in low-risk group, of which MHC class I pathway was the highest infiltration (Figure 8(d) and 8(f)).

### 2.9. The Correlation of Drug Sensitivity and 10-PRG Signature

**Figure 9:**
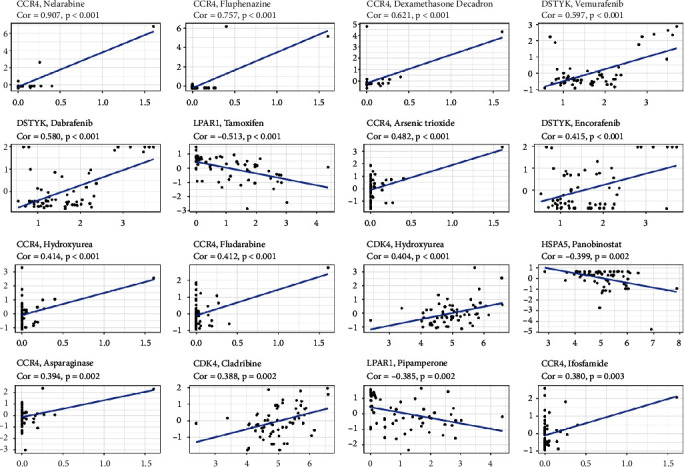
Correlations between cell risk score and IC50 for different drugs. *X*-axis is each gene expression and *Y*-axis is the *Z* score (IC50) of each drug.

To better connect our PRG signatures to clinical practice, paraptosis-based signature was utilized to filter these compounds that were collected from CellMiner database to determine the drug sensitivity. Only 153 drugs that Food and Drug Administration (FDA) has authorized to apply were involved to explore the correlation between 10-PRG signature and half of inhibited concentrationand the result was shown that 85 drugs with significantly diffrence were identified (Table S1).  Figure 9 displays the most relevant top 16 correlations to paraptosis-based signature. Specifically, CCR4 has more relations with the drug IC50, such as sensitive to nelarabine, fluphenazine, dexamethasone decadron, arsenic trioxide, hydroxyurea, fludarabine, asparaginase, and ifosfamide. The drug-resistance of tamoxifen and pipamperone increased with the upregulation of LPAR1. In addition, the drug sensitivity of vemurafenib, dabrafenib, and encorafenib increased with upregulation of DSTYK (all *P* < 0.05; Figure 9).

## 3. Discussion

Paraptosis, as an atypical form of programmed cell death, that has been manifested in recent years is playing a crucial role in anti-cancer mechanism of various natural products by mediating in tumor cell death, thereby providing a novel and prospective thought that can help find the therapeutic target in cancer research [25]. Paraptosis could also mediate through factors, such as oxidative stress, tumor microenvironment, and exosomes, which are closely related to occurrence and development of LGG. In recent years, research of tumor microenvironment participating in glioma invasion has made progress. It is reported that the increasing glioma-associated macrophages/microglia release lots of factors to degrade the extracellular matrix and provoke signaling pathways to promote glioma cell invasion [26]. In the process of oxidative stress, paraptotic cell death was induced by ROS with cytotoxicity produced by macrophage in glioma cells, overproduction of ROS, and reactive nitrogen species enhanced the infiltration of macrophages in tumor cells, promoting the proliferation and invasion of gliomas cells [27, 28]. This suggested that macrophages could change the tumor microenvironment to promote cell invasion and mediate the paraptosis by oxidative stress in glioma cells. Extracellular vesicles have multiple functions in the central nervous system and are closely related to the communication between cell types within glioblastoma and their microenvironment [29]. Glioblastoma-induced exosomes can increase the oxidative stress of cerebellar neurons by reducing cellular antioxidant defense and increasing oxidative damage [30]. Lai et al.'s study found that thrombospondin-1 can improve the hypoxia-induced paraptosis through regulating exosome protein expression in human corneal epithelial cells [31]. Nevertheless, whether glioma–exosomes could mediate paraptosis to function remains unknown.

Noteworthily, the certain form of cell death that resemble paraptosis was found in neurodevelopment and degeneration found; therefore, understanding the biochemical pathways of paraptosis has potential implications for probing neurodegeneration, cancer treatment, development, and evolution of cell death procedures [11]. However, little knowledge was found in the connection between paraptosis and the development and evolution of LGG. In this present study, we first explored the prognostic accuracy of PRGs for LGG patients via bioinformatics analysis. By cluster analysis based on DE-PRGs, three clusters with differences in survival were found in the LGG patients. This provides a brand-new cognition for understanding the classification of PRGs via the consensus clustering analysis in LGG patients. To further explore the role of paraptosis in LGG, we performed LASSO and multivariate Cox regression model to identify 10-paraptosis-related signatures. In addition, this risk score and nomogram prognostic model show excellent performance for predicting the development of LGG. The immune-related infiltration, biological processes, and pathways were enriched in PRGs of LGG, such as antigen presentation processes, MHC biological processes, and highly infiltrated in the cluster 1 and high-risk group.

In our study, we developed successfully a prognostic model from TCGA, GEO, and CGGA based on 10-PRG signatures (CDK4, TNK2, DSTYK, CDKN3, CCR4, CASP9, HSPA5, RGR, LPAR1, and PDCD6IP). Specifically, high levels of CDK4 were observed in glioma tissues, and the inhibitors of CDK4/6 block cell proliferation, induced apoptosis, and enhanced the cell sensitivity to temozolomide in glioma patients [32]. Besides, high CDKN3 mRNA levels commonly occurred and were associated with poor OS in a variety of human cancer cells, such as LGG, renal clear cell carcinoma, and prostate adenocarcinoma [33]. In addition, CDKN3 was also observed as a hazard factor in this prognostic model of LGG patients. In addition, TNK2 could stimulate PDGFR-*β* activity and AKT activation to promote tumor cell cycle progression, proliferation, and tumorigenesis and played a pivotal role in PDGFR-induced AKT signaling in glioma tumorigenesis [34]. DSTYK is participated in the activation of NF-*κ*B, JNK, and p38 pathways and induction of apoptosis; DSTYK induces cell death through caspase-dependent and caspase-independent pathways [35, 36]. CCL2 recruits immunosuppressive regulatory T cells that express CCR4 to induce Tregs migration to glioma tissue for immune evasion, which is the main sign of tumorigenesis and a powerful obstacle to effective cancer treatment in gliomas [37]. Surprisingly, Maru et al. discovered that the expression of CCR4 was decreased in glioma cells compared with adult human astrocytes and might have a latent role in glioma cell proliferation [38]. Caspase 9 (CASP9), a member of caspase family, mediates paraptosis induction using an IGF1R, although paraptosis is a programmed cell death caspase-independent [11, 39]. Experiments have demonstrated that CASP9 displays at least two distinct activities that are not only pro-apoptosis but also non-apoptosis cell death [39]. Song et al. found that ivermectin could induce apoptosis and paraptosis by increasing the activity of CASP3, CASP9, and blocked cell cycle in G0/G1 phase, by downregulating the expression of CDK and cyclin levels in glioma cells [40]. HSPA5 localizes to the lumen of ER, involving several cellular processes, such as polypeptide transport, folding, and assembly of protein [41]. In addition, the upregulating protein levels of HSPA5 were mainly responsible for the paraptotic changes associated with ER dilation of breast cancer cells [42]. Taken together, DSTYK, CASP9, and HSPA5 are the key catalysts of paraptosis induction, whereas other signatures are involved in regulating the process of paraptosis that may reserve a questioning attitude. However, the roles of other signatures in LGG remain unknown, and the specific mechanism of gene interaction needs to be explored.

Enrichment analysis displayed that PRGs are involved in multiple signal pathways, especially in immune-related pathways, suggesting that PRGs play a vital role in immune infiltration and are hoped to be the potential markers. In ssGSEA analysis, the immune status was apparently different when comparing low- and high-risk groups. Immune cells and pathways are widely considered as some of the most significant proponents of anti-cancer in recent years [43]. In our study, an immunosuppressive microenvironment was revealed in high-risk LGG patients, such as the inhibition of NK cells and more active Treg and tumor macrophages [44]. However, the higher levels of crucial anti-tumor infiltrating immune statuses were also found in the high-risk group. The reason for this difference may be that glioma cells have immune evasion feature. Decrease of neoantigen expression was also reported to be relevant with the inhibited immune function to develop immune evasion and influence the efficacy of immunotherapy in glioma [45]. A repertoire of inhibitory checkpoint ligands that regulate effector T cell responses is expressed in the glioma cells. In addition, glioblastoma cells with several immune inhibitory checkpoint ligands inhibited the major T cell checkpoint receptors to suppress the immune function [46]. Notably, our study also revealed that the check-point pathway was more active in high-risk LGG patients. Immunotherapy approaches may become effective ways to improve the anti-tumor elements in LGG patients. Besides, Chen et al. found that the T9 glioma cells producing the macrophage colony stimulating factor can be killed by the macrophages based on paraptosis [19]. Moreover, the paraptotic cells can induce the release of heat shock protein and high mobility group B-1 signals by activating the big potassium channel, which strongly activates the antigen presentation process and thus enhances anti-tumor immunity and enables non-genetically modified tumor vaccines to become an accessible project based on the paraptosis process in glioma cells [47]. High-risk LGG patients showed more active macrophages microenvironment in our study. Therefore, activating paraptosis may be an efficient path to inhibiting immune evasion in LGG patients.

Even though our model showed a better prognostic effect for LGG patients, this study still had some limitations. First, the collection of all data was downloaded from publicly available databases, forming the retrospective research. Second, enrichment analysis uncovered the great correlation in the PRGs and immune-related processes, but the specific mechanism is suspicious. Third, our study only conducted computational analysis and still further needs to be validated and explored by experiments. Thus, prospective cohort study and relevant PRG experiments should be undertaken to support our results in the future.

In summary, this study found that three different paraptosis clusters exist in LGG patients, and they are associated significantly with OS. We first conducted a prognostic model based on PRGs to investigate the connection between PRGs and OS in the LGG patients, via a comprehensive bioinformatics analysis. Our analysis proved that this risk model had a greater predictive consequence to predict the prognosis of LGG patients based on TCGA, CGGA, and GEO. Our findings provide a novel knowledge of paraptosis status and a crucial principle for further exploring the role of PRGs signature in LGG.

## 4. Materials and Methods

### 4.1. Data Acquisition and Preprocessing

**Figure 10:**
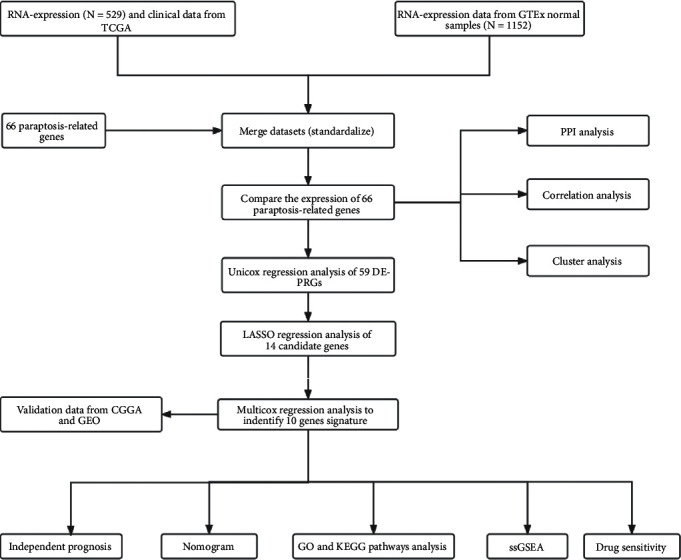
Flowchart of the study.

The RNA sequencing expressions with the fragments per kilobase per million format of 529 LGG patients and their clinical information with BCR xml format were acquired from TCGA database (https://portal.gdc.cancer.gov/). The RNA-seq expressions of 1152 normal brain samples were procured from GTEx database (https://xenabrowser.net/datapages/). The RNA-seq expressions and their clinical data of 443 LGG patients were obtained for external validation from the mRNAseq_693 dataset of CGGA database (http://www.cgga.org.cn/), a professional database about glioma. The above data acquisition process based on TCGA and CGGA databases was completed on 21 August 2021. The dataset of GSE16011 from GEO database was acquired for another external validation, comprising 107 samples after including completed survival information (https://www.ncbi.nlm.nih.gov/geo/, accessed on 17 October 2022) [48]. All the datasets used were freely available online in the above databases. All gene expression data from TCGA and GTEx databases were normalized to eliminate the influence of batch effect by using “limma” and “sva” R packages. Additionally, in TCGA and CGGA cohorts, the clinical data incomplete and incongruent would be excluded in the selection of datasets. The clinical characteristics including age, gender, grade, histology, and survival status of TCGA and CGGA cohorts were included to analyze in our research (Table S2). The overall research flow of our study was displayed in Figure 10. After browsing a large number of relevant documents, 66 PRGs were extracted from the published studies in PubMed database and were listed in Table S3.

### 4.2. Recognition of DE-PRGs and Their Interaction Network

To avoid the omission of key candidate genes, we set the criteria of *P* < 0.05 to detect the DE-PRGs with the “limma” and “pheatmap” packages, between 529 tumor samples and 1152 normal samples. PPI network for 66 PRGs was conducted using the Search Tool for the Retrieval of Interacting Genes website (https://string-db.org/, accessed on 9 September 2021), setting an interaction score of 0.7 and hidden disconnected nodes. The correlation heatmap for DE-PRGs was also analyzed and drawn using the “corrplot” package.

### 4.3. Consensus Clustering Analysis

The clustering analysis was performed to distinguish paraptosis-related model to the marker genes with prognostic value based on univariate Cox method. Consensus matrix (*k* = 2–9) was set with “ConsensusClusterPlus” package. The matrix with better distinguishable was selected to classify in TCGA and CGGA cohorts, respectively. Moreover, PCA and OS-related analyses were conducted to verify whether there were distinct clustering and classification. Immune infiltration in paraptosis cluster including the ESTIMATE score, immune score, and stromal score was also evaluated by the “ESTIMATE” package. Next, 7 algorithms, including TIMER, CIBERSORT, CIBERSORT-ABS, QUANTISEQ, MCPCOUNTER, XCELL, and EPIC, were adopted to compare the concentration of immune cells in different clusters.

### 4.4. Development of the Prognostic Model and External Validation

After normalization for TCGA, CGGA, and GEO cohorts, further analyses were performed as follows. A univariate Cox regression analysis was utilized to screen the prognostic PPGs using *P* < 0.01 as the filtering criteria. A forest plot with *P* value, hazard ratio, and 95% confidence interval was formed using “survival” package. LASSO Cox regression analysis was further used to narrow the candidate genes with “glmnet” package, and the penalty parameter was determined when the partial likelihood deviance was at its minimum. Subsequently, based on these candidate PRGs, the multivariate Cox regression was applied to establish the prognostic model and determine the signature genes, and the regression coefficient of each significant prognostic-genes was then calculated. In this model, the computational formula for the risk score: risk score = ∑_*n*=1_^*N*^coefficient(*i*) × expression(*i*), was utilized to acquire the risk score of each sample, where *N* is represented as the number of the signature genes and *i* is represented as each significant prognostic-genes. According to the median risk score, LGG patients were separated into low- and high-risk subgroups. The OS of both the low- and high-risk subgroups was compared. We conducted the PCA and *t*-SNE analyses using “rtsne” package to observe risk classification. Furthermore, time-dependent ROC was performed to predict the performance of the prognostic risk model based on survival time of patients in 1, 3, and 5 years. In TCGA cohort, OS-related analysis and AUC were judged for accuracy and feasibility of the prognostic risk model. The above results were also evaluated in CGGA and GEO cohorts to verify this risk model. Besides, we also performed the OS-related analysis for each significant signature in this model based on the GEPIA2 (http://gepia2.cancer-pku.cn/, accessed on 6 November 2021) database that is an online tool to work some basic bioinformatics analyses.

### 4.5. Independent Prognostic Analysis and Construction of a Predictive Nomogram

The clinical features were processed to further investigate the prognostic values, using Cox regression analysis for TCGA and CGGA cohorts. Subsequently, a nomogram was employed to predict the OS of 1, 3, and 5 years based on multifactor comprehensive analysis using “rms” package. Based on CGGA and TCGA cohorts, the distribution difference between actual survival probability and predicted survival probability was calibrated by repeating the sampling for 1000 times, and the *C*-index method was used to verify the efficiency of the nomogram.

### 4.6. Functional Enrichment and Drug Sensitivity Analyses

GO analysis was used by loading “clusterProfiler”, “http://org.hs.eg.db”, “enrichplot”, and “ggplot2” packages, with the criteria of *P* < 0.05. Similarly, KEGG pathway analysis was also conducted to discover the main pathways of execution for paraptosis-induced. Then, we executed the ssGSEA to explore immune status in LGG via “GSVA” and “GSEABase” packages. The data for GO and KEGG analyses was accessed on 14 September 2021. The boxplots listed the scores of immune cells infiltration and the activity of immune-related pathways based on low- and high-risk groups in TCGA and CGGA. The IC50 of over 20,000 chemical compounds was obtained from the CellMiner database (https://discover.nci.nih.gov/cellminer/home.do accessed on 21 August 2021). Among them, only FDA-approved anti-tumor drugs were eligible to analyze the relation between paraptosis-based risk score and drug sensitivity. Spearman's correlation analysis with *P* < 0.05 was conducted to estimate their correlation.

### 4.7. Statistical Analysis

Data analysis and visualization were operated using, and the corresponding R packages were executed in the corresponding analysis. The Wilcoxon test was utilized to access the difference between the normal and tumor sample variables for identification of DE-PRGs. The Kruskal–Wallis test was used to compare the division of tree clusters in the immune infiltration analysis. Meanwhile, the chi-square test was also utilized to find the differences in the clinical category, and the Spearman's analysis calculated the correlation coefficient. Kaplan–Meier analysis with log rank test was implemented to evaluate the significant difference between two subgroups in OS. The *C*-index is used to estimate the probability that the predicted result is consistent with the actually observed result and to evaluate the prediction ability of the model. The Mann–Whitney *U* test was exploited to obtain the GSEA scores of immune cells and immune pathways between the low- and high-risk groups.

## Data Availability

The data could be download at https://portal.gdc.cancer.gov/, https://xenabrowser.net/, https://www.cgga.org.cn/, https://gepia2.cancer-pku.cn/, https://www.ncbi.nlm.nih.gov/geo/, and https://discover.nci.nih.gov/cellminer/home.do, and the relevant original data used during the current study are available from the corresponding author on a reasonable request.

## Supplementary Materials

Supplementary Materials Table S1 The correlation results found on drug IC50 and 10-PRGs signature. Table S2 Clinical information of TCGA and CGGA cohorts. Table S3 66 paraptosis-related genes. Figure S1 Prognostic assessment of 10-PRG signatures.
