# Supplementary material for: A Novel Insight into Paraptosis-Related Classification and Signature in Lower-Grade Gliomas
Source: Int J Genomics. 2022 Nov 14;2022:6465760. doi: 10.1155/2022/6465760 (PMC9678488; doi:10.1155/2022/6465760)
Supplement: Supplementary Materials — Table S1 The correlation results found on drug IC50 and 10-PRGs signature. Table S2 Clinical information of TCGA and CGGA cohorts. Table S3 66 paraptosis-related genes. Figure S1 Prognostic assessment of 10-PRG signatures. [file 6465760.f1.docx]

Table S1 the correlation between 10-PRG signature and half of inhibited concentration.

| Gene | Drug | cor | *P* value |
| --- | --- | --- | --- |
| CCR4 | Nelarabine | 0.906733 | 2.04E-23 |
| CCR4 | Fluphenazine | 0.756606 | 2.71E-12 |
| CCR4 | Dexamethasone Decadron | 0.621387 | 1.17E-07 |
| DSTYK | Vemurafenib | 0.597449 | 4.67E-07 |
| DSTYK | Dabrafenib | 0.579662 | 1.22E-06 |
| LPAR1 | Tamoxifen | -0.5132 | 2.75E-05 |
| CCR4 | Arsenic trioxide | 0.482253 | 9.55E-05 |
| DSTYK | Encorafenib | 0.415282 | 0.000969 |
| CCR4 | Hydroxyurea | 0.414313 | 0.000998 |
| CCR4 | Fludarabine | 0.411922 | 0.001075 |
| CDK4 | Hydroxyurea | 0.404327 | 0.001355 |
| HSPA5 | Panobinostat | -0.39948 | 0.001567 |
| CCR4 | Asparaginase | 0.394386 | 0.00182 |
| CDK4 | Cladribine | 0.387639 | 0.002213 |
| LPAR1 | Pipamperone | -0.38474 | 0.002403 |
| CCR4 | Ifosfamide | 0.380343 | 0.00272 |
| RGR | 6-Thioguanine | -0.3797 | 0.00277 |
| LPAR1 | Ixabepilone | -0.37655 | 0.003024 |
| LPAR1 | Raloxifene | -0.37227 | 0.003401 |
| LPAR1 | Ifosfamide | -0.3715 | 0.003473 |
| CCR4 | Irofulven | -0.37043 | 0.003575 |
| LPAR1 | Lomustine | -0.36909 | 0.003708 |
| LPAR1 | DAUNORUBICIN | -0.36381 | 0.004271 |
| CDK4 | Chlorambucil | 0.362113 | 0.004468 |
| DSTYK | Docetaxel | -0.3602 | 0.0047 |
| CCR4 | DACARBAZINE | 0.357591 | 0.005031 |
| CDK4 | Fludarabine | 0.355082 | 0.00537 |
| CCR4 | Cyclophosphamide | 0.349686 | 0.006168 |
| LPAR1 | Crizotinib | -0.34593 | 0.006783 |
| CDK4 | Uracil mustard | 0.344458 | 0.007038 |
| CDK4 | Cytarabine | 0.341776 | 0.007524 |
| CCR4 | Chlorambucil | 0.340775 | 0.007713 |
| CCR4 | Pipobroman | 0.339235 | 0.008012 |
| CDK4 | Floxuridine | 0.337977 | 0.008264 |
| CASP9 | TYROTHRICIN | 0.337881 | 0.008283 |
| LPAR1 | Acrichine | -0.33727 | 0.008407 |
| CDK4 | Vinorelbine | -0.33339 | 0.009239 |
| CDK4 | Triethylenemelamine | 0.331932 | 0.009571 |
| TNK2 | TYROTHRICIN | 0.331687 | 0.009627 |
| LPAR1 | Oxaliplatin | -0.33048 | 0.00991 |
| LPAR1 | Carmustine | -0.32921 | 0.010215 |
| LPAR1 | Arsenic trioxide | -0.32874 | 0.010331 |
| LPAR1 | Celecoxib | -0.32398 | 0.011561 |
| CDK4 | Melphalan | 0.323398 | 0.01172 |
| DSTYK | ARRY-162 | 0.32298 | 0.011835 |
| TNK2 | Vemurafenib | 0.32078 | 0.012458 |
| CDK4 | Gemcitabine | 0.320326 | 0.01259 |
| CDK4 | Thiotepa | 0.319408 | 0.01286 |
| CDK4 | 7-Ethyl-10-hydroxycamptothecin | 0.318363 | 0.013174 |
| CCR4 | Melphalan | 0.317713 | 0.013373 |
| LPAR1 | Cyclophosphamide | -0.31728 | 0.013508 |
| CCR4 | Calusterone | 0.316824 | 0.013649 |
| CDK4 | Vorinostat | 0.31183 | 0.01529 |
| CDK4 | tfdu | 0.3095 | 0.016113 |
| CDK4 | Irinotecan | 0.308854 | 0.016347 |
| LPAR1 | Dasatinib | 0.308729 | 0.016393 |
| RGR | Dasatinib | -0.30802 | 0.016654 |
| LPAR1 | Homoharringtonine | -0.3073 | 0.016922 |
| PDCD6IP | Vinorelbine | -0.30728 | 0.016929 |
| DSTYK | ABT-199 | 0.306857 | 0.017091 |
| PDCD6IP | JNJ-42756493 | -0.30682 | 0.017105 |
| CCR4 | Carmustine | 0.305644 | 0.017557 |
| CDK4 | Nelarabine | 0.304929 | 0.017836 |
| DSTYK | Selumetinib | 0.304554 | 0.017984 |
| PDCD6IP | Eribulin mesilate | -0.3043 | 0.018084 |
| CDK4 | Parthenolide | 0.304171 | 0.018136 |
| HSPA5 | Neratinib | -0.30254 | 0.018798 |
| LPAR1 | Vincristine | -0.29814 | 0.020683 |
| CCR4 | Oxaliplatin | 0.297241 | 0.021089 |
| HSPA5 | Allopurinol | -0.29577 | 0.021766 |
| CCR4 | Trametinib | -0.29426 | 0.022478 |
| CCR4 | Idarubicin | 0.293947 | 0.02263 |
| DSTYK | brigatinib | -0.29372 | 0.022741 |
| LPAR1 | Vorinostat | -0.29354 | 0.022828 |
| LPAR1 | LDK-378 | -0.29344 | 0.022877 |
| CCR4 | Uracil mustard | 0.292476 | 0.023348 |
| CDKN3 | LEE-011 | 0.291195 | 0.023989 |
| CCR4 | Etoposide | 0.290526 | 0.02433 |
| CCR4 | Cytarabine | 0.290522 | 0.024332 |
| CDK4 | Pemetrexed | 0.290438 | 0.024375 |
| TNK2 | Tegafur | 0.289877 | 0.024665 |
| CCR4 | 6-THIOGUANINE | 0.287649 | 0.025842 |
| CCR4 | DECITABINE | 0.287204 | 0.026082 |
| LPAR1 | DECITABINE | -0.2842 | 0.027757 |
| CDKN3 | Denileukin Diftitox Ontak | -0.28393 | 0.027911 |
| CDK4 | Pipobroman | 0.283408 | 0.028214 |
| CDK4 | Dexamethasone Decadron | 0.282407 | 0.0288 |
| DSTYK | Ixazomib citrate | 0.282186 | 0.028931 |
| CCR4 | Thiotepa | 0.281574 | 0.029296 |
| LPAR1 | Irofulven | 0.279942 | 0.030287 |
| HSPA5 | 6-THIOGUANINE | -0.27986 | 0.030335 |
| CDK4 | Clofarabine | 0.279639 | 0.030474 |
| DSTYK | Cobimetinib (isomer 1) | 0.279518 | 0.030549 |
| CCR4 | Digoxin | 0.278908 | 0.030929 |
| CCR4 | Lomustine | 0.27635 | 0.032567 |
| DSTYK | Palbociclib | -0.27496 | 0.033487 |
| LPAR1 | TESTOLACTONE | -0.27296 | 0.034851 |
| LPAR1 | Dromostanolone Propionate | -0.27236 | 0.035265 |
| HSPA5 | Lapatinib | -0.27211 | 0.035438 |
| PDCD6IP | Fluorouracil | -0.27205 | 0.035484 |
| CDKN3 | Temsirolimus | -0.27192 | 0.035572 |
| CCR4 | Cobimetinib (isomer 1) | -0.27176 | 0.035685 |
| LPAR1 | Epirubicin | -0.27122 | 0.03607 |
| TNK2 | Erlotinib | -0.27114 | 0.036129 |
| HSPA5 | Bosutinib | -0.26971 | 0.037162 |
| DSTYK | Dasatinib | -0.26945 | 0.037347 |
| LPAR1 | Erlotinib | 0.26866 | 0.037932 |
| CASP9 | JNJ-42756493 | -0.26859 | 0.037987 |
| LPAR1 | DACARBAZINE | -0.26652 | 0.039546 |
| CCR4 | Triethylenemelamine | 0.265932 | 0.040004 |
| CCR4 | Dexrazoxane | 0.264947 | 0.040774 |
| HSPA5 | 6-MERCAPTOPURINE | -0.26489 | 0.040816 |
| LPAR1 | Panobinostat | -0.26344 | 0.041973 |
| TNK2 | Neratinib | -0.26337 | 0.042031 |
| CDK4 | 6-THIOGUANINE | 0.262873 | 0.042437 |
| CDK4 | Topotecan | 0.2619 | 0.043236 |
| LPAR1 | Isotretinoin | -0.26173 | 0.043377 |
| PDCD6IP | brigatinib | -0.2611 | 0.043904 |
| CASP9 | 6-Mercaptopurine | -0.26104 | 0.043956 |
| RGR | 6-THIOGUANINE | -0.26046 | 0.044439 |
| PDCD6IP | Paclitaxel | -0.25774 | 0.046794 |
| LPAR1 | Vinblastine | -0.25731 | 0.047172 |
| CASP9 | brigatinib | -0.25652 | 0.047883 |
| CASP9 | Palbociclib | -0.25602 | 0.048327 |
| CDK4 | 6-Thioguanine | 0.254828 | 0.049421 |

Table S2 Clinical information of TCGA and CGGA cohorts.

|  | TCGA cohort (n=513) | | CGGA cohort (n=442) | |
| --- | --- | --- | --- | --- |
| Variable | Number/total | %/total | Number | %/total |
| Age |  |  |  |  |
| <=20 | 10 | 1.95% | 9 | 2.04% |
| 21-40 | 243 | 47.37% | 219 | 49.55% |
| 41-60 | 199 | 38.79% | 198 | 44.80% |
| >60 | 61 | 11.89% | 16 | 3.62% |
| Gender |  |  |  |  |
| Female | 229 | 44.64% | 192 | 43.44% |
| Male | 284 | 55.36% | 250 | 56.56% |
| Grade |  |  |  |  |
| G2 | 248 | 48.34% | 188 | 42.53% |
| G3 | 265 | 51.66% | 254 | 57.47% |
| Histology |  |  |  |  |
| Astrocytoma | 194 | 37.82% | 271 | 61.31% |
| Oligoastrocytoma | 129 | 25.15% | 141 | 31.90% |
| Oligodendroglioma | 190 | 37.04% | 30 | 6.79% |

Table S3 66 paraptosis-related genes

| Genes | Full-names |
| --- | --- |
| CAMK2B | Calcium/calmodulin dependent protein kinase II beta |
| PRKACG | Protein kinase cAMP-activated catalytic subunit gamma |
| MARK4 | Microtubule affinity regulating kinase 4 |
| SSTR5 | Somatostatin receptor 5 |
| TAAR5 | Trace amine associated receptor 5 |
| USP10 | Ubiquitin specific peptidase 10 |
| PRKAG3 | Protein kinase AMP-activated non-catalytic subunit gamma 3 |
| HACD2 | 3-hydroxyacyl-CoA dehydratase 2 |
| NT5C | 5', 3'-nucleotidase, cytosolic |
| INSRR | Insulin receptor related receptor |
| SSTR3 | Somatostatin receptor 3 |
| TAAR9 | Trace amine associated receptor 9 |
| HSPB8 | Heat shock protein family B (small) member 8 |
| PLPP2 | Phospholipid phosphatase 2 |
| G6PC2 | Glucose-6-phosphatase catalytic subunit 2 |
| GUCY2EP | Guanylate cyclase 2E, pseudogene |
| CDK4 | Cyclin dependent kinase 4 |
| RGR | Retinal G protein coupled receptor |
| ADGRG1 | Adhesion G protein-coupled receptor G1 |
| UQCRC1 | Ubiquinol-cytochrome c reductase core protein 1 |
| TNK2 | Tyrosine kinase non receptor 2 |
| RNF181 | Ring finger protein 181 |
| MKNK2 | MAPK interacting serine/threonine kinase 2 |
| UBE2U | Ubiquitin conjugating enzyme E2 U |
| MYLK | Myosin light chain kinase |
| CTDSP2 | CTD small phosphatase 2 |
| LCK | LCK proto-oncogene, Src family tyrosine kinase |
| GPR15 | G protein-coupled receptor 15 |
| ATP23 | ATP23 metallopeptidase and ATP synthase assembly factor homolog |
| LPAR1 | Lysophosphatidic acid receptor 1 |
| PI4KB | Phosphatidylinositol 4-kinase beta |
| DSTYK | Dual serine/threonine and tyrosine protein kinase |
| CFD | Complement factor D |
| PPP3CA | Protein phosphatase 3 catalytic subunit alpha |
| CCR4 | C-C motif chemokine receptor 4 |
| PRAG1 | PEAK1 related, kinase-activating pseudokinase 1 |
| CDKN3 | Cyclin dependent kinase inhibitor 3 |
| GPR153 | G protein-coupled receptor 153 |
| DDIT3 | DNA damage inducible transcript 3 |
| MAPK8 | Mitogen-activated protein kinase 8 |
| MAP2K2 | Mitogen-activated protein kinase kinase 2 |
| MAPK1 | Mitogen-activated protein kinase 1 |
| MAPK14 | Mitogen-activated protein kinase 14 |
| IGF1R | Insulin like growth factor 1 receptor |
| PDCD6IP | Programmed cell death 6 interacting protein |
| CASP9 | Caspase 9 |
| ERN1 | Endoplasmic reticulum to nucleus signaling 1 |
| ATF6 | Activating transcription factor 6 |
| XBP1 | X-box binding protein 1 |
| AKT1 | AKT serine/threonine kinase 1 |
| EIF2S1 | Eukaryotic translation initiation factor 2 subunit alpha |
| HSPA5 | Heat shock protein family A (Hsp70) member 5 |
| CASP4 | Caspase 4 |
| CASP3 | Caspase 3 |
| CASP7 | Caspase 7 |
| ITPR3 | Inositol 1,4,5-trisphosphate receptor type 3 |
| RYR1 | Ryanodine receptor 1 |
| RYR2 | Ryanodine receptor 2 |
| MCU | Mitochondrial calcium uniporter |
| TNFRSF19 | TNF receptor superfamily member 19 |
| PDCD5 | Programmed cell death 5 |
| CSF1 | Colony stimulating factor 1 |
| TP53 | Tumor protein p53 |
| NFKB1 | Nuclear factor kappa B subunit 1 |
| PEBP1 | Phosphatidylethanolamine binding protein 1 |
| PHB | Prohibitin |

Related genes from the following references:

1. Kim JY, Lee DM, Woo HG, Kim KD, Lee HJ, Kwon YJ, Choi KS. RNAi Screening-based Identification of USP10 as a Novel Regulator of Paraptosis. Sci Rep. 2019 Mar 20;9(1):4909. doi: 10.1038/s41598-019-40982-z. PMID: 30894572; PMCID: PMC6427038.
2. Sperandio S, Poksay K, de Belle I, Lafuente MJ, Liu B, Nasir J, Bredesen DE. Paraptosis: mediation by MAP kinases and inhibition by AIP-1/Alix. Cell Death Differ. 2004 Oct;11(10):1066-75. doi: 10.1038/sj.cdd.4401465. PMID: 15195070.
3. Garrido-Armas M, Corona JC, Escobar ML, Torres L, Ordóñez-Romero F, Hernández-Hernández A, Arenas-Huertero F. Paraptosis in human glioblastoma cell line induced by curcumin. Toxicol In Vitro. 2018 Sep;51:63-73. doi: 10.1016/j.tiv.2018.04.014. Epub 2018 Apr 30. PMID: 29723631.
4. Yoon MJ, Kim EH, Lim JH, Kwon TK, Choi KS. Superoxide anion and proteasomal dysfunction contribute to curcumin-induced paraptosis of malignant breast cancer cells. Free Radic Biol Med. 2010 Mar 1;48(5):713-26. doi: 10.1016/j.freeradbiomed.2009.12.016. Epub 2009 Dec 28. PMID: 20036734.
5. Yumnam S, Hong GE, Raha S, Saralamma VV, Lee HJ, Lee WS, Kim EH, Kim GS. Mitochondrial Dysfunction and Ca(2+) Overload Contributes to Hesperidin Induced Paraptosis in Hepatoblastoma Cells, HepG2. J Cell Physiol. 2016 Jun;231(6):1261-8. doi: 10.1002/jcp.25222. Epub 2015 Dec 2. PMID: 26492105.
6. Wang Y, Li X, Wang L, Ding P, Zhang Y, Han W, Ma D. An alternative form of paraptosis-like cell death, triggered by TAJ/TROY and enhanced by PDCD5 overexpression. J Cell Sci. 2004 Mar 15;117(Pt 8):1525-32. doi: 10.1242/jcs.00994. PMID: 15020679.
7. Hoa NT, Zhang JG, Delgado CL, Myers MP, Callahan LL, Vandeusen G, Schiltz PM, Wepsic HT, Jadus MR. Human monocytes kill M-CSF-expressing glioma cells by BK channel activation. Lab Invest. 2007 Feb;87(2):115-29. doi: 10.1038/labinvest.3700506. PMID: 17318194.
8. Li B, Zhao J, Wang CZ, Searle J, He TC, Yuan CS, Du W. Ginsenoside Rh2 induces apoptosis and paraptosis-like cell death in colorectal cancer cells through activation of p53. Cancer Lett. 2011 Feb 28;301(2):185-92. doi: 10.1016/j.canlet.2010.11.015. Epub 2010 Dec 30. PMID: 21194832.
9. Wang CZ, Li B, Wen XD, Zhang Z, Yu C, Calway TD, He TC, Du W, Yuan CS. Paraptosis and NF-κB activation are associated with protopanaxadiol-induced cancer chemoprevention. BMC Complement Altern Med. 2013 Jan 3;13:2. doi: 10.1186/1472-6882-13-2. PMID: 23281928.
10. Sperandio S, Poksay KS, Schilling B, Crippen D, Gibson BW, Bredesen DE. Identification of new modulators and protein alterations in non-apoptotic programmed cell death. J Cell Biochem. 2010 Dec 15;111(6):1401-12. doi: 10.1002/jcb.22870. PMID: 20830744.


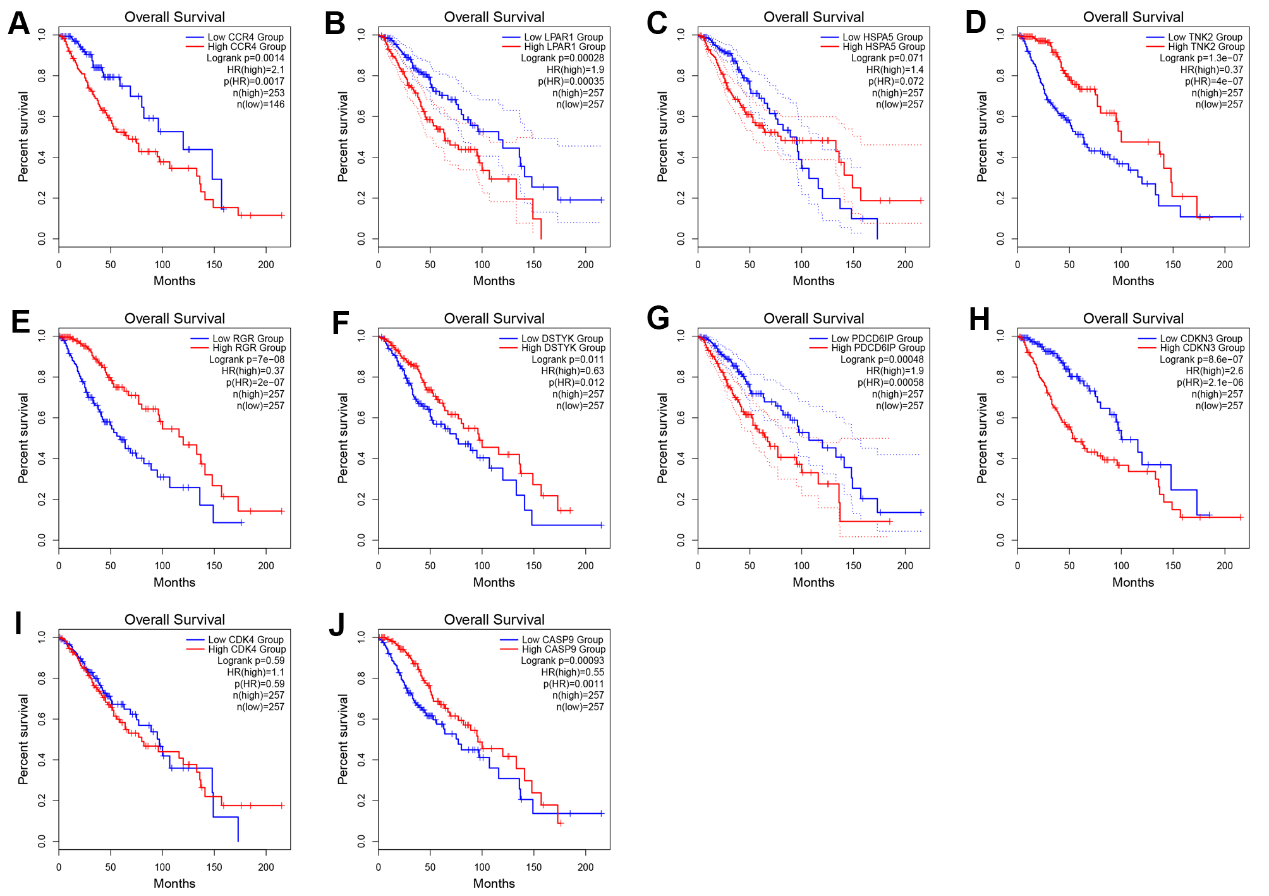


Figure S1 Prognostic assessment of 10-PRG signatures.
